# Femur first surgical technique: a smart non-computer-based procedure to achieve the combined anteversion in primary total hip arthroplasty

**DOI:** 10.1186/s12891-017-1688-9

**Published:** 2017-08-01

**Authors:** Mattia Loppini, Umile Giuseppe Longo, Emanuele Caldarella, Antonello Della Rocca, Vincenzo Denaro, Guido Grappiolo

**Affiliations:** 1 Hip Diseases and Joint Replacement Surgery Unit, Humanitas Clinical and Research Centre, Via Alessandro Manzoni 56, 20089 Rozzano, Milan Italy; 2 Humanitas University, Via Alessandro Manzoni 113, 20089 Rozzano, Milan Italy; 3 Department of Orthopaedic and Trauma Surgery, Campus Bio-Medico University, Via Alvaro del Portillo 200, 00128 Trigoria, Rome Italy

**Keywords:** Combined anteversion, Acetabular inclination, Femur first, Hip, Arthroplasty

## Abstract

**Background:**

The relevance of prosthetic component orientation to prevent dislocation and impingement following total hip arthroplasty (THA) has been widely accepted. We investigated the use of a non-computer-based surgery to address the reciprocal orientation of the acetabular and femoral components.

**Methods:**

In the femur first technique, the cup is positioned relative to the stem. When the definitive antetorsion of femoral component is fixed, the cup is positioned in a compliant anteversion to the stem. Clinical and radiographic assessments were performed before and 3 months after THA. Radiographic assessment was performed in standing position with the EOS 2D/3D radiography system. 3D images were used to preoperative anterior pelvic plane (APP) angle, postoperative acetabular inclination (AI) and anteversion (AA), and postoperative stem antetorsion. Clinical assessment was performed with Harris Hip Score (HHS).

**Results:**

Forty patients (40 hips) underwent primary THA with an average age of 61 years (range, 36–84). Average HHS increased from 43 ± 5 (range, 37–52) preoperatively to 97 ± 6 (range, 86–100) at the last follow-up (*P* < 0.0001). Average combined anteversion value of cup with liner and stem was 38° ± 9° (range, 12°-55°). Average AI value of cup with liner was 39° ± 6° (range, 30°-55°) in the group with standard stem and 45° ± 7° (range, 39°-58°) in the group with varized stem (*P* = 0.007). Relationship analysis showed no correlation between the combined anteversion values of the cup with liner and stem with APP angle values (*r* = 0.26, *P* = 0.87).

**Conclusions:**

Femur first technique allows the surgeon to achieve a combined anteversion ranging from 25° to 50° with a cup inclination ranging from 30° to 50°. The cup is positioned according to the functional plane of the patient regardless the preoperative pelvic tilt.

## Background

Over the last two decades, a growing relevance has been recognized to the prosthetic component orientation to prevent dislocation and impingement following total hip arthroplasty (THA).

In 1978, Lewinnek et al. [1] identified the orientation of the acetabular cup associated with the lower rate of dislocation. The suggested “safe zone” was included between 30°-50° of inclination in the coronal plane, and 5°-25° of anteversion in the axial plane. Although malpositioning of the acetabular component has been demonstrated to affect significantly the range of motion (ROM), joint stability, wear and loosening [2–5], the relative orientation of the acetabular and femoral components seems to be as important as the absolute positioning based on the bony landmarks.

The concept of combined anteversion was introduced by Ranawat et al. [6] in 1991, suggesting that the sum of the cup anteversion and stem antetorsion should be 45° for women and between 20° and 30° for men. Subsequently, several mathematical models were developed to determine the combination of cup inclination and anteversion, and stem antetorsion providing the greater ROM and lower risk of cup-neck impingement [7–9]. Among these studies, the authors recommended a cup inclination between 40° and 45°, combined with cup anteversion and stem antetorsion determined by the following formula: cup anteversion + (0.7 x stem antetorsion) = X°, where the value of X ranged from 37° to 42°. On the other hand, clinical use of combined anteversion has determined it should be between 25° up to 50° [10].

Combined anteversion technique demonstrated to reduce of 6 times the dislocation rate in cementless total hip arthroplasty [11]. Some authors suggested the intraoperative navigation to achieve a proper combined anteversion [10, 12]. In this respect, computer-based systems can detect malpositioning of the first component, and correct accordingly the second component to restore the reciprocal orientation of both components. However, the setup and application of conventional navigation systems are time- and cost-consuming, and smart non-computer-based devices should be developed to allow the surgeon to achieve the proper combined anteversion [13].

In the present study, we investigated a non-computer-based surgical technique to obtain a proper orientation of both acetabular and femoral components during a primary THA procedure, according with the concept of combined anteversion. In the femur first technique, the cup is positioned relative to the stem after the trial stem has been implanted [14]. Therefore, the definitive antetorsion of femoral component is fixed, and the cup is positioned in a compliant anteversion to the first component. Moreover, because of a relation between cup inclination and the neck-to-shaft angle of straight stems has been described [15], we also hypothesized that the femur first technique could provide the coverage of the cup accordingly with the neck-to-shaft angle of the stem. Finally, because the present technique is based on the combined anteversion concept, we hypothesized that proper functional orientation of both cup and stem components should not be affected by the preoperative value of pelvic tilt.

Although previous studies evaluated the effectiveness of femur first technique to achieve a proper combined anteversion [10, 11, 16], none investigated its role to determine the coverage of the cup, and the relationship of the combined anteversion values with pelvic tilt. The null hypotheses of the study were: 1) the femur first technique did not allow the surgeon to achieve a combined anteversion ranging from 25° to 50°, and a cup inclination ranging from 30° to 50°; and 2) there was no correlation between preoperative pelvic tilt and postoperative combined anteversion values if the femur first technique is performed.

## Methods

### Sample

Forty patients (40 hips) who underwent primary total hip arthroplasty from November 2014 to February 2015 were enrolled. Patients included 17 men and 23 women, with an average age of 61 years (range, 36–84) at the time of the index procedure. The average body mass index at the surgery was 27 kg/cm^2^ (range, 18–39). The preoperative diagnosis included: primary osteoarthritis in 33 patients, osteoarthritis secondary to mild development dysplasia of the hip in 6 patients, and post-trauma osteoarthritis in one patient. Exclusion criteria included: patients eligible for partial or total THA revision, THA associated with other procedures (i.e. femoral osteotomy), previous pelvic and/or femoral osteotomy, severe hip dysplasia (Crowe III or IV), primitive or metastatic tumors of hip joint, previous spine and/or sacroiliac joint instrumentation, previous or current hip joint infection.

A minimum follow-up of 1 year was achieved in all patients.

### Surgical procedure

In all patients, the digital preoperative radiograph in AP view was used to perform preoperative planning with the software Hip Arthroplasty Templating 2.4.3 running with OsiriX v.5.8.1 64-bit [17].

Surgery was performed by one experienced surgeon with the patient in the lateral position, through a posterolateral approach in all cases. After the release of short external rotators tendons at the insertion to greater trochanter, the capsule was incised, and the femur was dislocated. After the neck osteotomy, the femur was prepared by holding the knee flexed with the tibia in a vertical position. The starter device was introduced with the planned version for the definitive stem. The femoral rasps were inserted gradually increasing the size until proper fit was achieved. The definitive antetorsion of the stem was related with the native anatomy of the proximal femur. The type of neck, standard or varized, was defined according with the native value of caput-collum-diaphyseal angle (CCD), as measured with the radiographic assessment.

Maintaining the proper sized rasp in situ to protect the femoral shaft, the acetabulum was exposed, the labrum was removed, and the acetabulum was sequentially reamed. The definitive position of the acetabular component, in terms of anteversion and inclination angle, was identified basing of femoral stem anteversion and CCD angle. The femur was reduced with the proper sized rasp in situ by using a 44 mm trial plastic head with a long neck to compensate the absence of the acetabular component. By using a large head, it was possible to avoid an eccentric position of the head in the native acetabulum. After reduction, with the hip in neutral position at 0° of flexion and abduction, the femur was internally rotated to obtain an angle of 35°, measured with a sterile goniometer, between the longitudinal axis of the tibia and the operating table which is parallel with the floor. Because of the degree of internal rotation to produce a coplanar head and cup is the combined anteversion [6], the definitive cup should be placed parallel with the horizontal line on the trial head in both axial and coronal plane. The parallelism in the axial plane provided the proper degree of cup anteversion to obtain a combined anteversion of 35°. On the other hand, the parallelism in the coronal plane provided the proper degree of cup inclination angle. The peripheral rim of the determined position of the cup was marked with a dermographic pen on the bony surface of the acetabulum. After the femur dislocation, the definitive acetabular cup was implanted, and osteophytes surrounding the acetabulum were removed when indicated. Then, the femur was reduced with the trial components in situ without the liner, and it was placed in internal rotation of 35° with the hip in neutral position at 0° of flexion and abduction. Therefore, the coplanarity of head and cup in both axial and coronal plane was checked, and the position of the liner with the elevated rim was determined in order to optimize the joint congruency. Previous joint dislocation, the femoral rasp was removed, the definitive liner was implanted with the elevated rim in planned position, and the definitive stem was implanted. Finally, the proper length of the neck was chosen to restore the native vertical and lateral offsets. At the end, the internal rotation with hip flexion and the free ROM were performed to assess the stability of the hip [18]. No impingement, dislocation and lower limb discrepancy were observed in any patient.

In all patients, uncemented cup and stem were implanted. The acetabular system was: G7 cup (Zimmer Biomet) in 31 hips and Trabecular Metal Acetabular Shell (Zimmer Biomet) in 9. The stem system was: GTS stem (Zimmer Biomet) in 23 hips and CLS stem (Zimmer Biomet) in 17. The stem was standard in 29 hips (21 GTS with CCD of 136° and 8 CLS with CCD of 135°) and varized in 11 (2 GTS with CCD of 123° and 9 CLS with CCD of 125°).

In all patients, the bearing surface consisted in ceramic head and polyethylene liner with 10° elevated rim. The location of the elevated rim of the liner was reported by using a clock-face description where 12 o’clock indicated the side towards the head of patient, and 6 o’clock was the side towards the obturator foramen [17]. To clarify the data presentation, the site of the elevated rim was standardized to the right hip. The location of the elevated rim of the liner was: 12 o’clock in 1, 11 o’clock in 12, 9 o’clock in 6, 7 o’clock in 17, and 3 o’clock in 4 hips.

### Clinical assessment

The Harris Hip Score (HHS) was performed to evaluate the clinical picture of the patient before and 3 months after surgery. The questionnaire assigns up to 91 points for the domains of pain and hip function, and up to 9 points for the domain of range of motion. The final score ranges from 0 to 100 points, with the higher scores indicating the better clinical picture. The HHS has been considered excellent for values between 90 and 100 points, good for values between 80 and 89, fair for values between 70 and 79, and poor for values under 70 [19].

The intraoperative and postoperative complications, and the occurrence of hip dislocation were also evaluated.

### Radiographic assessment

All patients had a radiographic assessment in standing position before and 3 months after THA with the EOS 2D/3D radiography system (Biospace Med, Paris, France).

EOS system allows to achieve an AP and lateral radiographic view of the whole skeletal system [20–22]. The 2D images were used to perform a 3D reconstruction of skeletal system and prosthetic components with a dedicated software (sterEOS 3D, versione 1.5.3.7947, Biospace Med, Paris, France). 3D images were used to measure preoperative CCD angle and femoral antetorsion [23], preoperative anterior pelvic plane (APP) angle, postoperative acetabular inclination (AI) and anteversion (AA), and postoperative stem antetorsion [24].

The CCD angle was defined as the angle between the femoral neck axis and femoral long axis [25]. The APP angle was defined as the angle subtented by a vertical reference line and a line tangent to the anterosuperior iliac spines and the pubic symphysis [22]. The combined anteversion was determined by the following formula: cup anteversion + (0.7 x stem antetorsion) [8]. The AI and AA were measured in the patient frame based on a vertical plane passing through the centre of the acetabular cup, which avoids the effect of a potential axial rotation of the pelvis during acquisition. On the other hand, the stem antetorsion was measured relative to posterior bi-condylar plane.

The AI and AA values measured with EOS system were also adjusted according the location of the liner’s elevated rim. The 12 o’clock position was considered to reduce the AI value of 10°. The 3 o’clock and 9 o’clock positions were considered to reduce and increase the AA value of 10°, respectively. Finally, the 11 o’clock and 7 o’clock positions were not considered able to influence AI and AA values as measured with EOS system.

The outliers were the values out of the targeted ranges of combined anteversion (from 25° to 50°) and AI (from 30° to 50°).

## Statistics

All the analyses were performed using SPSS for Mac (version 23.0, SPSS Inc., Chicago, Illinois). Descriptive statistics were calculated. The categorical variables were expressed as frequency with percentage. Continuous variable data were expressed as mean with standard deviation and range as minimum and maximum values.

The Wilcoxon signed-rank test with two tails was used to compare the preoperative and postoperative values of HHS. It was also used to compare the preoperative femoral antetorsion and postoperative stem antetorsion. The U Mann-Whitney test with two tails was used to compare the AI and combined anteversion values measured without liner and values recorded taking account the location of elevated rim of the liner. It was also used to compare the AI values of cup with liner in the group with standard stem and those in the group with varized stem. Finally, it was used to compare the combined anteversion values of the cup with liner and stem in the group with antetorsion of the stem ≤9° and those in the group with ≥10° of antetorsion of the stem.

The Spearman rank correlation matrix was used to assess the relationship between the combined anteversion values of the cup with liner and stem with APP angle values. For this relationship, a linear regression model was also performed and the coefficient of determination was calculated (R^2^). The P was considered significant for values less than 0.05.

## Results

### Clinical outcome

The average HHS increased from 43 ± 5 (range, 37–52) preoperatively to 97 ± 6 (range, 86–100) at the last follow-up (*P* < 0.0001). The final scores were good in 13, and excellent in 27.

No patients reported intraoperative or postoperative complications neither dislocation of the prosthesis at the last follow-up.

### Radiographic outcome

**Fig. 1:**
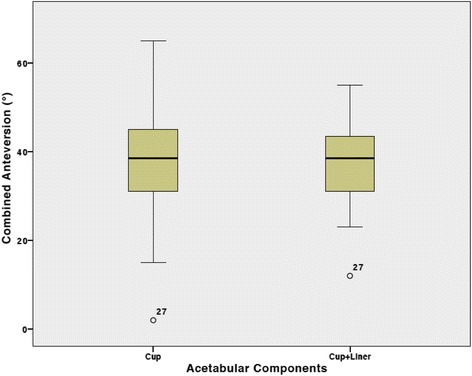
Box plots showing the combined anteversion values measured in the cup with and without liner groups. Angles are expressed in degrees

The average combined anteversion value with and without taking account the location of elevated rim of the liner was 38° ± 9° (range, 12°-55°) and 37° ± 13° (range, 2°-65°) respectively (*P* = 0.88). In the cup with liner group, there were 3 (7.5%) outliers up to 13°, whereas there were 11 (27.5%) outliers up to 23° in the cup without liner group (Fig. 1).

**Fig. 2:**
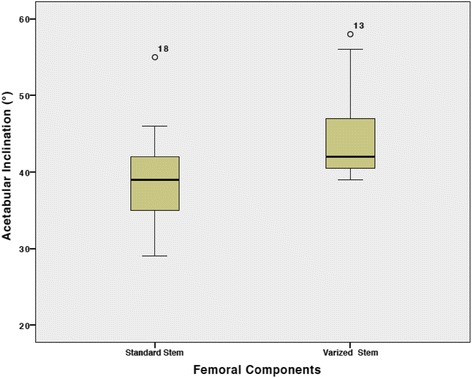
Box plots showing the acetabular inclination values measured in the standard stem and varized stem groups. Angles are expressed in degrees

The average AI value with and without taking account the location of elevated rim of the liner was 40° ± 6° (range, 30°-58°) and 41° ± 7° (range, 30°-58°) respectively (*P* = 0.8). In both groups, there were 3 (7.5%) outliers up to 8°. In the subgroup analysis, the average AI value of cup with liner was 39° ± 6° (range, 30°-55°) in the group with standard stem and 45° ± 7° (range, 39°-58°) in the group with varized stem (*P* = 0.007) (Fig. 2).

**Fig. 3:**
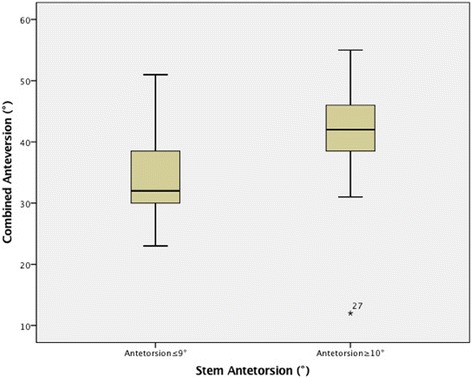
Box plots showing the combined anteversion values measured with the cup-liner system in the group with ≤9° and ≥10° of antetorsion of the stem. Angles are expressed in degrees

The average preoperative femoral antetorsion was 11° ± 14° (range, 21° of retrotorsion - 45° of antetorsion), whereas the average postoperative stem antetorsion was 9° ± 10° (range, 23° of retrotorsion - 31° of antetorsion) (*P* = 0.71). The average combined anteversion value of the cup with liner and stem was 34° ± 8° (range, 23°-51°) in the group with antetorsion of the stem ≤9° and 41° ± 9° (range, 12°–55°) in the group with ≥10° of antetorsion of the stem (*P* = 0.002) (Fig. 3). Thirteen out of 19 (68%) patients with a preoperative femoral antetorsion ≥10° reported a postoperative antetorsion of the stem ≥10° as well.

**Fig. 4:**
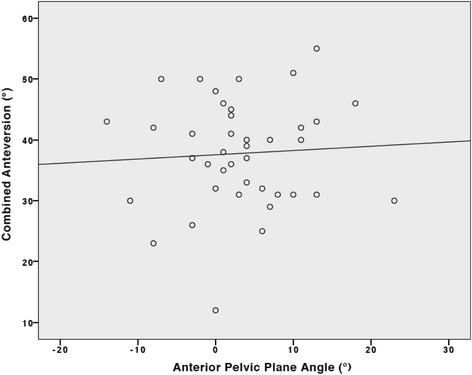
Scatter plot between combined anteversion values measured with the cup-liner system and anterior pelvic plane values. Linear regression: y = 37.55 + 0.07 * x (R^2^ = 0.004, *P* = 0.71)

The relationship analysis showed no correlation between the combined anteversion values of the cup with liner and stem with APP angle values (*r* = 0.26, *P* = 0.87) and no linear regression between these variables (R^2^ = 0.004, *P* = 0.71) (Fig. 4).

## Discussion

In the present study, we rejected the first null hypothesis and we accepted the second null hypothesis: 1) the femur first technique allowed to achieve a combined anteversion ranging from 25° to 50° with a cup inclination ranging from 30° to 50°; and 2) there was no correlation between preoperative pelvic tilt and postoperative combined anteversion values.

About the first hypothesis, the combined anteversion value was within the targeted range in 92.5% of hips with an average value of 38° ± 9°. The results achieved with our non-computer-based procedure were similar to those reported by Dorr et al. [10] with intraoperative computer navigation. The authors found a combined anteversion within the targeted range from 25° to 50° in 96% hips with an average value of 35.9° ± 6.7°. On the other hand, Nakashima et al. [11] reported with their manual technique a combined anteversion within the targeted range from 40° to 60° in 73% hips with an average value of 50.3 ± 6.2°. Although the manual placement of the cup was associated with 27% of outliers, the combined anteversion technique significantly reduced the dislocation after primary THA.

In our current practice, we prefer to use a liner with 10° elevated rim if polyethylene is implanted to increase the hip stability [26, 27]. Therefore, we would suggest to use the elevated rim to improve the joint congruency even if the combined anteversion is within the targeted range. In our series, in 29 (72.5%) hips, the elevated rim was not used to change the combined anteversion and it was placed in the posterosuperior quadrant (11 o’clock) or posteroinferior quadrant (7 o’clock) to further increase the hip stability in internal rotation or flexion of the femur respectively. In the remaining hips (27.5%) we used the elevated rim to improve the combined anteversion as required by the intraoperative assessment with the femur at 35° of internal rotation. Because there was no impingement or hip subluxation or dislocation, the position of the cup was not changed in these patients, but the combined anteversion was improved by positioning the elevated rim anteriorly (3 o’clock) or posteriorly (9 o’clock).

The analysis of the combined anteversion values according with the antetorsion values of the stem showed that the combined anteversion values in the group with ≥10° of stem antetorsion were significantly higher than those in the group with stem antetorsion ≤9° (*P* = 0.002). These findings demonstrated that the combined anteversion is strongly affected by the femoral antetorsion suggesting morphotype plays a critical role in the position of the definitive prosthetic components.

In our series, the average postoperative stem antetorsion was 9° ± 10° ranging from 23° of retrotorsion to 31° of antetorsion. These results are similar to those reported in previous studies. Dorr et al. [10] showed with the computer navigation a wide range of positions of uncemented femoral stems ranging from 17° of retroversion to 28° of antetorsion. Pierchon et al. [28] and Wines et al. [29] used a postoperative CT scans reporting a range from 30° of retroversion to 45° of antetorsion of both cemented and uncemented stems. The wide range of position of the stem in terms of torsion is related with the high variability in the geometry of the proximal femur. Because the stem should have the best fit in the bone its position is affected by the patient-specific femoral anatomy.

In our series, we reported an AI value within the targeted range in 92.5% of hips with an average value of 40° ± 6°. Although we reported 3 outliers, two of them were included in the group with varized stem. The range of AI from 30° to 50° suggested by Lewinnek et al. [1] was not related with the value of CCD angle of the stem. However, we believe that cup inclination should be congruent with the neck of the stem [15]. For this reason, we would suggest to use the femoral component to determine intraoperatively the proper AI. The definitive cup should be placed parallel with the horizontal line on the trial head in both axial and coronal plane. Thus, the surgeon is able to provide the coverage of the cup according with the CCD angle of the femoral stem. Our results demonstrated that the group with varized stem had AI values significantly higher than those reported in the group with standard stem (*P* = 0.007). In this respect, the two outliers of 56° and 58° respectively are acceptable values if associated with a varized stem. In the present study, we did not evaluate the effect of pelvic obliquity in the frontal plane on postoperative AI. However, because of the AI was determined by the CCD angle of the femoral stem, we believe that postoperative AI is not affected by pelvic obliquity.

About the second hypothesis, the combined anteversion value achieved with femur first technique was not affected by preoperative pelvic tilt. Because of the anteversion and inclination of the cup are strongly related with the patient-specific pelvic tilt and spine sagittal balance [20, 30], some authors suggested to use intraoperative navigation taking into account the preoperative pelvic tilt of the patient [31–34]. The “safe zone” suggested by Lewinnek et al. [1] was referred to bony landmarks assuming no tilt of the pelvis. However, in the most of patients the pelvic tilt is not neutral. For this reason, the navigation systems referring to anatomical bony landmarks need to consider the pelvic tilt and correct for their measurements to avoid inaccurate position of the cup. In the present technique, the surgeon did not need to consider the pelvic tilt. The patient was in the lateral position with the pelvis hold without change the patient-specific pelvic tilt and sagittal balance. After the femoral component was fixed in terms of antetorsion and CCD angle, the cup was positioned in a compliant fashion to the stem with the hip in neutral position at 0° of flexion and abduction. Therefore, the cup was positioned according to the stem without tacking into account the preoperative pelvic tilt. In this respect, the cup was positioned according to the functional plane without changing the patient-specific pelvic tilt and spinal sagittal balance. Indeed, our results demonstrated that preoperative APP angle values did not correlate with the combined anteversion values after surgery.

During a THA procedure, the vast majority of surgeons place the prosthetic cup with the native acetabular anteversion and then place the stem achieving the best fit in the femur. Disadvantages of this approach include higher risk of hip dislocation and reduced ROMs due to a suboptimal combined anteversion [35, 36]. To overcome these complications, some authors advocated the use of femur first technique to obtain an optimal combined anteversion. Dorr et al. [10] proposed to perform a cementless THA preparing the femur first with the estimation of antetorsion, and then implanting the cup with computer navigation. Authors recommended a mean combined anteversion of 35° (range, 25° to 45°). On the other hand, Unlu et al. [16] proposed a femur first procedure using the lesser trochanter as a landmark to estimate the femoral component antetorsion. They suggested to estimate intraoperatively the antetorsion of the femoral component by the formula: operative collo-trochanteric angle (defined as the angle between the collo-femoral axis and the lesser trochanter axis) minus 34° (mean lesser trochanteric version). Finally, the position of the cup should be adjusted to provide a mean combined anteversion of 37° (range, 25° to 50°) by internal rotation without hip flexion with patient in lateral decubitus. Finally, Nakashima et al. [11] proposed to measure the stem antetorsion intraoperatively with a goniometer as the angle between the perpendicular to the axis of the tibia hold in vertical position and the neck of the stem. The cup was positioned with 20° of anteversion by using the manufacturer’s jig and then it was adjusted according the stem antetorsion to achieve the targeted combined anteversion.

Although previous studies evaluated the effectiveness of femur first technique to achieve a proper combined anteversion [10, 11, 16], to our knowledge, this is the first study demonstrating that a non-computer-based femur first technique could be also used to obtain a proper inclination of the acetabular cup according with the CCD angle of the stem. Indeed, previous studies focused only to achieve a proper combined anteversion trough the femur first procedure. Moreover, we also demonstrated that the postoperative combined anteversion values achieved with this technique are not affected by preoperative APP angle values.

We are aware that the present study is affected by some limitations. First, this case series had no control cases who underwent primary THA with conventional surgical procedure. Therefore, we were not able to ascertain the superiority of femur first technique over a conventional procedure characterized by the implantation of the cup first according anatomical bony landmarks. Further studies should be performed to address this comparison. Second, all patients were operated by a single experienced surgeon. Therefore, further studies should be performed to compare in standardized fashion the outcomes achieved by junior and senior surgeons with this technique. However, in our current practice, this procedure is easily performed by all surgeons of the unit with reliable results. Third, the fixation of uncemented stem requires a stable press fit into the bone, and the implant must adapt to the variable femoral geometry. Therefore, to achieve the desired stem antetorsion could be difficult, and the present findings of antetorsion may not be referred to all commercial designs. For this reason, only two straight femoral stems were used. Moreover, the optimum range for combined anteversion is still controversial. In clinical setting, some authors proposed a range from 25° to 50° [10, 16], whereas others suggested a range form 40° to 60° [11, 37]. The present technique may allow the surgeon to adjust the orientation of the cup basing on stem antetorsion in order to provide a reliable combined antiversion within the range between 25° to 50°. Forth, we used a liner with 10° elevated rim and the definitive combined anteversion was based on the anteversion of the cup-liner system and stem. However the position of the elevated rim was collected in a systematic fashion and the radiographic measurements of AA were corrected for. Fifth, we did not compare the intraoperative estimation of the stem antetorsion by the surgeon with the postoperative radiographic measurements. However, previous studies demonstrated the low accuracy of surgeon’s estimation of the stem torsion. Dorr et al. [10] reported that intraoperative estimation by the surgeon had outliers between 6° to 10° and than 10° in 23% of the hips respectively. Hirata et al. [38] demonstrated that the average value of error for the surgeon’s intraoperative estimation of the stem anteversion was 7.3° ranging from 11° underestimation to 25° overestimation. Moreover, the aim of the study was to evaluate the combined anteversion and AI values achieved with the femur first procedure.

## Conclusions

The femur first technique is a non-computer-based procedure that allows the surgeon to achieve a combined anteversion ranging from 25° to 50° with a cup inclination ranging from 30° to 50°. Moreover, the cup is positioned according to the functional plane of the patient regardless the preoperative pelvic tilt.

## Abbreviations

AA: acetabular anteversion
AI: acetabular inclination
APP: anterior pelvic plane
CCD: caput-collum-diaphyseal
HHS: Harris Hip Score
ROM: range of motion
THA: total hip arthroplasty
